# Gaining insights into pet owner understanding/lived experience of canine chronic kidney disease using survey and social media data

**DOI:** 10.3389/fvets.2025.1506272

**Published:** 2025-05-07

**Authors:** Georgina Tarrant, Luke Boyden, Taranpreet Rai, Andrea Wright, Alasdair J. C. Cook, Kevin Wells

**Affiliations:** ^1^Surrey DataHub, vHive, School of Veterinary Medicine, University of Surrey, Guildford, United Kingdom; ^2^Centre for Vision Speech and Signal Processing, University of Surrey, Guildford, United Kingdom; ^3^Outcomes Research, Zoetis, Loughlinstown, Dublin, Ireland

**Keywords:** canine chronic kidney disease, canine CKD, social media analysis, large language models, AI, survey data, canine chronic renal failure, canine CRF

## Abstract

**Introduction:**

Chronic kidney disease (CKD) is a common and progressive condition in dogs characterized by irreversible damage to one or both kidneys over an extended period leading to gradual decline in kidney function. Early diagnosis is crucial to improve quality of life and increase survival through medical interventions.

**Methods:**

This study investigated pet owner understanding of this condition using insights gained by comparing pet owner survey responses with bulk harvested social media discussions on canine CKD. We combined structured survey data (*n* = 132) with social media analysis spanning multiple platforms to understand owner perceptions of disease characteristics, clinical sign reporting, and pet owner experiences.

**Results:**

Both data sources highlighted increased urination and water consumption as primary pet owner concerns, with these clinical signs showing moderate positive correlation (Pearson correlation coefficient of *r* = 0.66). Although not explicitly investigated within the survey, social media data demonstrated pain as a significant concerning clinical sign and revealed the emotional toll of end-of-life care considerations. Further analysis also demonstrated significant associations between CKD diagnosis in dogs and both animal age (*p* < 0.001) and female gender (*p* = 0.006), while breed group and weight showed no significant correlations.

**Discussion:**

The complementary nature of structured surveys and social media analysis provided richer understanding of pet owner experiences, understanding and management of CKD. This combined methodological approach offers a model for investigating other chronic conditions in veterinary medicine.

## 1 Introduction

Chronic kidney disease (CKD) is a common clinical condition in small animals, particularly affecting senior dogs. The disease is characterized by gradual, irreversible damage to one or both kidneys over a period exceeding three months, leading to decreased kidney function or resulting from sequential acute kidney injury episodes (1–3). CKD is said to be the most common disease in dogs but current estimates of CKD prevalence vary widely from 0.05 to 3.74% depending on the source dog population and case inclusion criteria (4). The disease is progressive, manifesting with clinical signs including polydipsia, polyuria, urinary incontinence, proteinuria, low body condition score, weight loss, vomiting, anorexia, diarrhea, anemia, hypertension, hypoalbuminemia, cough, lethargy (1, 5) and pain in acute cases (1).

Current treatment protocols focus on clinical sign management and slowing disease progression, but significant challenges remain in early diagnosis and disease monitoring. The underlying disease etiology of CKD can be wide ranging making it difficult to diagnose during clinical investigation; irreversible kidney impairment often occurs before diagnosis (1). Early diagnosis of CKD allows for more effective medical interventions to maintain a higher quality of life (QoL) and increased survival (2, 3). Although incurable, ongoing disease management can prolong a dog’s life for months or years (2, 5, 6). Effective management requires close collaboration between veterinarians and pet owners to monitor patient response to treatment, encourage long-term compliance with treatment plans, prevent pet owner disengagement and avoid premature euthanasia (3).

Despite advancements in the management of canine CKD, significant knowledge gaps remain in our understanding of the mechanisms driving CKD development and progression (1, 3, 4, 6–9). We seek to support understanding in this area through the first joint analysis of both dog owner survey and social media (SM) data exploring pet owner knowledge of CKD, observed clinical signs and key challenges in managing affected dogs, and to thus better understand the lived experience and disease understanding of pet owners managing dogs with CKD.

Traditional data collection methods (surveys and focus groups) each present their own limitations. The conventional method of gathering data on companion animal veterinary disease is via pet owner surveys, which are quick and easy to deliver but may also introduce potential biases (through question selection and presentation style) and have limited sampling (8). Focus groups and interviews provide detailed qualitative data but may not capture the full spectrum of experiences (6, 9). Alternatively, analysis of social media data although providing unsolicited information free from question bias, may potentially contain sample bias from online platform-specific posting behavior (10–13). Thus, all sampling methods carry potential drawbacks which need to be considered in any subsequent analysis. Social media listening (SML) presents an alternative approach to gaining insights into pet owner perceptions from harvesting large amounts of publicly available online narratives (14). Commercial or open-source web scraping tools can collect this data, with subscription-based services such as Pulsar Platform™ being fully licensed to harvest or ‘scrape’ data from numerous platforms (15). AI and machine learning tools then offer opportunities to analyze bulk harvested SML data for insights into, for example, veterinary healthcare and personalized medicine.

The objective of this study was to investigate the use of traditional survey methods and social media data analysis to enhance our understanding of canine CKD from the pet owner perspective and thus inform the development of future surveys. The primary research question was: “What insights on canine chronic kidney disease can be gained by comparing pet owner survey responses with social media discussions?”

To address this, we examined several secondary research questions:

- What are the demographic characteristics of dogs diagnosed with CKD in our study population?
- What clinical signs do pet owners most commonly observe and find most concerning in dogs with CKD?
- How do clinical sign reports differ between structured surveys and spontaneous social media discussions?
- Are pet owners willing to contribute data for ongoing research on canine CKD?

The study involved three key methodological steps:

- Conduct a survey to gather structured data on CKD in dogs
- Use survey findings to inform analysis of social media data streams
- Analyze and compare survey and social listening data

By examining both structured survey responses and unprompted social media discussions, this study aims to provide a comprehensive view of how pet owners experience and understand canine CKD. This combined approach allows us to identify patterns in the characteristics of affected dogs while capturing the broader context of pet owners’ real-world experiences and concerns.

## 2 Methodology

### 2.1 Ethical considerations

This study was reviewed by the University of Surrey Ethics Committee and then granted favorable ethical opinion on 15th April 2024, reference FHMS 21–22,026 EGA – Amend 2.

### 2.2 Survey design, administration, and participant selection

An online survey investigating CKD in dogs was designed by veterinarians with experience of canine CKD to support the subsequent development of a conceptual framework for a quality-of-life measure for dogs. Survey questions were informed by a manual literature review of current academic research on canine CKD, with veterinary review at each iteration before the survey was launched. A copy of the survey can be found in section 2.3 of the Supplementary materials. Further demographic information was provided based on the survey respondent’s dog’s user profile on the Pet Parade mobile application. All profile information is self-reported by users.

The survey was conducted from August 2023 to January 2024 using the Pet Parade mobile application, owned by Good Boy Studios (GBS). Pet Parade is a social platform where pet owners share photographs and videos for competitions and market research purposes. This was used to reach English-speaking dog owners in the US and UK to explore pet owner understanding around CKD. Survey participants were recruited from existing app users through in-app advertisements. Only participants who reported having a living dog diagnosed with CKD (by a veterinarian) were asked to complete the full survey, receiving a $10 Amazon voucher as an incentive. Additional information including the dog’s weight, breed and home country was collected from the user profile. The survey asked pet owners to identify the clinical signs their dog was experiencing and, from the same list of clinical signs, indicate which were most troubling for the dog and the pet owner when providing care. While “clinical signs” is a term that describes observable health indicators that animals cannot self-report, the term “symptoms” was used throughout the survey due to it being a familiar term to pet owners. Data from Pet Parade users with healthy dogs (without CKD) served as a control group, identified as those who answered “no” when asked if their dog had been diagnosed with CKD by a veterinarian. Further detail can be found in section 1.4 of the Supplementary materials.

### 2.3 Survey data analysis

The survey data underwent rigorous cleaning to ensure data quality. Survey responses (*n* = 1,633) were extracted from Pet Parade by GBS, who created a summary report and delivered this in addition to the full dataset. The dataset included dog profile information (as entered by the pet owner) in addition to survey responses. The survey data was subject to automated deduplication using Python to remove any duplicates or special characters and look for missing data. Manual spot checks were performed at each stage to validate the data cleaning pipeline’s effectiveness.

#### 2.3.1 Quantitative survey data analysis

We also performed statistical analysis on the survey data to provide further insights on CKD management. Descriptive statistics were generated from the survey data and these focused on dog characteristics, including weight and age (where available), country and state. These included Chi-squared tests to examine associations between CKD status and categorical demographic factors (age groups, gender, breed group, and weight profile). For all statistical tests, significance was established at *p* < 0.05. For highly significant results, *p* < 0.001 was used as the threshold. Results with *p* > 0.05 were considered not statistically significant. Analysis of variance (ANOVA) correlation tests were performed to investigate the relationships between CKD status and continuous variables (age, weight) (16). Logistic regression models were used to assess how these demographic factors predicted CKD presence and correlation analysis examined the co-occurrence patterns among reported clinical signs to identify potential clinical sign clusters.

To investigate whether dog age is correlated with the presence of CKD, we analyzed the survey data relating to dogs with CKD, dogs without CKD but with another kidney or renal condition and healthy dogs. The dogs were divided into seven age groups and then further divided into CKD and non-CKD dogs. The age groups chosen for analysis were: 0–2, >2–4, >4–6, >6–8, >8–10, >10–12, >12–14 and > 14–16 years. These intervals were chosen to easily interface with data provided from the app. Data for dogs more than 16 years of age was truncated as there were insufficient data points required to reliably perform a Chi-squared test.

The dog breeds reported in the survey were assigned to the equivalent American Kennel Club (AKC) breed group prior to statistical analysis using zero-shot classification (17–19). There are seven AKC breed groups: Sporting Group, Hound Group, Working Group, Terrier Group, Toy Group, Non-Sporting Group and Herding Group. Additional groups were used for mixed breed dogs and where the breed was not recognized. AKC breed groups were used in preference to the UK Kennel Club breed groups as the majority of survey respondents were in the US and although there are some similarities between the two, it was felt that the analysis would be clearer if only one set of breed groups was used. ANOVA correlation tests were used to examine potential correlations between dog breed / breed group and CKD status. The aim of the analysis was to determine whether any of the breed groups were significantly over- or under-represented in having CKD. The analysis controlled for gender, age and weight.

Weight profiles were qualitatively categorized on the app as: Swimsuit Model, Just Right and Overweight, with Overweight representing a combination of the Pet Parade app’s Lovingly Chubby and True Fatty categories due to low sample size in the latter (*n* = 2). These qualitative categories account for healthy and unhealthy weight variations across breed groups and enabled Chi-Square analysis of weight profiles and CKD probability. The analysis controlled for age, gender, and breed. The correlation between this weight percentile and CKD was then examined.

Chi-Squared tests were used to determine whether there was a significant difference between the expected and observed frequencies in the survey for dog age, AKC breed group, weight profile and gender. These tests assessed whether associations existed between variables, with significance determined at a level of *p* = 0.05. Key considerations for the analysis included ensuring a large enough sample size (with expected frequencies greater than 5), independent observations and mutually exclusive and exhaustive categories. For example, to ensure significance we only included AKC breed groups with at least 5 data points in both the healthy dogs’ group and the dogs with kidney conditions.

Logistic regression models were used to assess how dog gender and age predicted CKD status. The logistic regression model calculated the log-odds of a dog having CKD based on its age and gender as predictor variables (20–22). Additionally, an ANOVA correlation analysis was used to examine pair-wise correlation of the list of clinical signs examined in the survey. The intention was to verify that none of the 20 clinical signs from the survey (see Table 1) were redundant, i.e., whether any clinical sign is frequently coincident with another clinical sign.

**Table 1:** Grouped clinical signs from the survey as used for SML data analysis.

Group	Clinical signs
Urine issues	Blood in urine
	Urinary tract infection
	Increased or excessive urination
Hydration issues	Increased or excessive water consumption
	Dehydration
Food issues	Unexplained weight loss or muscle mass
	Loss or decrease in appetite
	Dog not eating at all
Energy level issues	Depressed mood
	Weakness or fatigue
	Sluggishness, lethargic
Stomach issues	Vomiting
	Diarrhea
	Stomach or intestinal inflammation
Mouth issues	Very bad breath
	Pale gums
	Mouth ulcers
Other issues	Problems with vision
	Messy appearance
	Fragile bones

### 2.4 Social listening methodology

To complement the survey data and gain additional insights into pet owners’ experiences and perceptions, a social media listening (SML) approach was employed. This may be thought of as analogous to large-scale open-ended focus group data using free form interview approaches. Data collection for this part of the study utilized passive listening using only publicly available data. No private groups or private data were used. We harvested social media data on CKD from posts across a multitude of social media platforms. While this is likely to be a different population sample to that used in the survey, it nonetheless represents a population heavily engaged in their dogs welfare.

**Figure 1:**
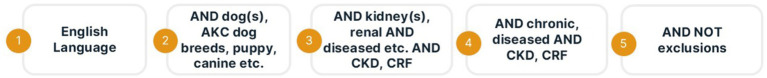
Example of the construction of a social media search expression to retrieve social media posts relevant to dogs that have been diagnosed with chronic kidney disease (CKD) from X/Twitter, Reddit, blogs and online forums. Only X required the retweet exclusion. Similar search expressions were developed for other platforms including Facebook and Pinterest. American Kennel Club (AKC).

The survey responses provided valuable insights into the everyday language and terminology used by pet owners when discussing canine CKD. These keywords and phrases were incorporated into the social listening strategy, allowing us to more effectively harvest and analyze online conversational data about CKD. Keyword search expressions using words and phrases carefully aligned with the survey questions were used, ensuring coherence and comparability between the two data sources as shown in Figure 1. The search expressions were deliberately of broad scope to facilitate potential discovery of novel clinical signs mentioned on social media but not in the survey. Selection of relevant social media sources and scoping searches to estimate potential search volumes across different social media platforms were devised and extracted using Pulsar Platform™. Keyword selections were optimized to represent more than 80% of relevant posts from a manually inspected sample of SML posts.

Data was collected from X (previously Twitter) for a five year period (5Y) from 1st January 2019 to 1st January 2024; from blogs, forums and Reddit for one year (1Y) from 1st January 2023 to 31 December 2023 and from all social media sources (X, blogs, forums, Reddit, Facebook and Pinterest) for a 30-day period (1M dataset) from 27th January 2024 to 25th February 2024. The variation in collection period was due to data scraping limitations dictated by different social media platform providers. Despite optimization of keyword searches as in Supplementary Figure S1, much of the data scraped from online platforms contains irrelevant, repetitive information that potentially adds noise to any subsequent analysis. It is therefore vital that data undergoes rigorous raw data cleaning and preprocessing steps to ensure data quality and relevance before extracting reliable and meaningful insights. To address this, social media posts that met our keyword requirement were first extracted from Pulsar Platform™, followed by automated deduplication using our own in-house software to remove exact matches and near-duplicates based on text similarity metrics. Although individual posts may describe the same animal multiple times, linking these references is difficult since social media data extraction processes each post in isolation, without preserving the contextual relationships between posts. The resulting cleaned dataset was then processed using natural language processing techniques to filter out noise (i.e., irrelevant content) such as advertisements and promotions, with manual spot checks performed to validate the data cleaning pipeline’s effectiveness.

**Figure 2:**
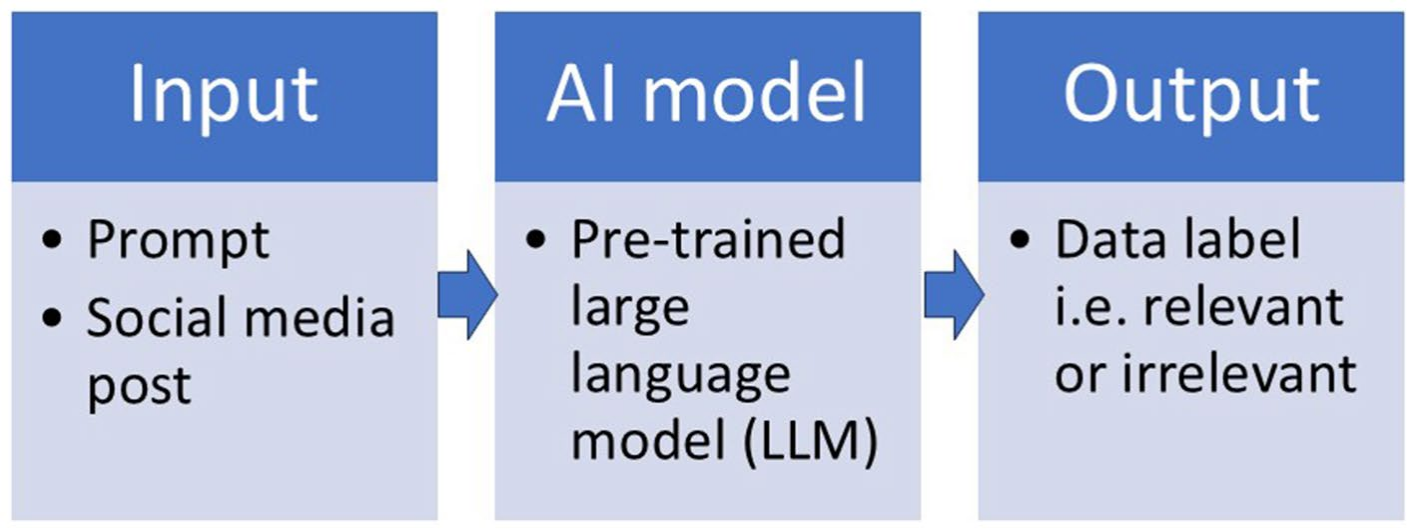
Zero-shot filtering schematic showing how extracted social media posts are classified as relevant or irrelevant to the research question by a pre-trained LLM.

Once cleaned, further irrelevant content was removed using Large Language Models (LLMs) to filter (classify) relevant social media posts via a zero-shot process (an LLM, such as ChatGPT), is a machine learning model that has been pre-trained on vast volumes of data. LLMs can recognize and generate text and perform other natural language processing tasks such as text classification (15). Zero-shot classification relies on an optimized prompt for the LLM that describes the task it is expected to perform and defines the desired outputs (18, 19). The LLM was prompted to label posts as relevant or irrelevant to the research questions using the steps shown in Figure 2. The prompt contained a detailed description for the LLM of what a relevant post would be (specifically focused on canine CKD) and what an irrelevant post would be (such as posts relating to human or feline CKD). This iterative approach involved progressively refining the input prompt for classifying relevance, beginning with broad initial classifications, which were then evaluated through manual spot-checks and adjusted through successive iterations. Regular manual checks comparing cleaned and classified data to the original scraped data were conducted throughout the process to ensure data quality and integrity.

### 2.5 Social listening data analysis

Descriptive statistics were generated from SML data, providing valuable insights into pet owners’ and dog demographics and characteristics. These additional SML insights were generated using demographic information provided by pet owners themselves and subsequent analysis using text-based sentiment analysis.

Posts were classified by sentiment using a bespoke in-house sentiment classifier to determine if a SM post was conveying a positive or negative sentiment regarding canine CKD. Our bespoke sentiment classifier had the advantage that it focused on the portions of a post relevant to a dog with CKD rather than classifying the sentiment of the whole post which could be lengthy and large parts of it being unrelated to CKD.

The clinical signs were grouped as shown in Table 1 to facilitate comparison with survey data (see section 2.6).

### 2.6 Comparative analysis between clinical sign reporting in SML and survey data

The SML findings were compared with the survey results, and further analysis was conducted to identify clinical signs reported online compared to those reported in the survey. This required the creation of dedicated logical expressions for each clinical sign to mine the data that had been scraped. For example, polydipsia (i.e., increased or excessive water consumption/thirst) was mined using the search expression:

(water OR drink OR drinking OR thirsty OR thirst OR fluid OR unquenchable OR dehydrated) AND NOT (“I’m thirsty” OR “I drink”).

As all posts had already been classified as pertaining to a dog with CKD, the grouped clinical sign expressions did not need to be restrictive. For example, if the word “water” appears in a post that has been confirmed as relating to a dog with CKD then it is reasonable to assume that the post mentions a dog drinking.

To facilitate statistical analysis, the 20 clinical signs from the survey were combined to form more general groups as shown in Table 1. If a survey respondent or social media poster specified any, some or all clinical signs within a given group this was only counted once, in order to make comparisons between the groups meaningful.

## 3 Results

### 3.1 Survey analysis

#### 3.1.1 Overview of survey and data quality

A survey advertisement was sent via the Pet Parade mobile app to 615,219 registered dog owners in the US and the UK. Only registered users of Pet Parade received the advertisement. The survey targeted pet owners with dogs of any age or breed. Of the 3,484 pet owners who viewed the survey, only 1,633 dog owners completed it. The first question asked whether the respondent’s dog was currently alive. A total of 358 responses were dropped as the pet owner said that their dog was not currently alive leaving 1,275 responses. Of these, 132 responses related to dogs with CKD (*n* = 97) or another kidney condition (*n* = 35), as diagnosed by a veterinarian. The remaining 1,143 dogs were considered healthy (not having CKD) based on their owners’ responses, including those who explicitly stated their dog did not have kidney disease and those who left the question blank. Therefore, we assumed that the total number of dogs owners who responded to the survey (*n* = 1,633) was representative of owners of dogs within the wider population.

Within the finalized survey dataset (*n* = 1,633), most respondents (96.9%) were from the US and 3.1% were from the UK. The gender split of dogs was near equal across the dataset with 50.7% male dogs compared to 49.3% female while the majority of dogs in the CKD group (*n* = 132) were female (*n* = 83, 62.9%). Small/toy dogs were most common in the full dataset (44.7%). Many were short-haired dogs (49.5%) as opposed to long haired (26.1%) or medium haired (24.4%). Of the responses regarding where the dog was adopted from, 25.5% answered friends or relatives, followed by breeder (25.3%), “other” (22.8%), animal shelters (19.4%), breeders (16.3%), pet stores (4.2%, US only), “other – rescued my pet” (1.9%), adoption website or app and “other – purchased my pet” (0.5% each). Although diet and hydration are crucial factors in managing CKD, surprisingly only 16 respondents in the CKD group provided information about their dog’s diet with 56.3% in this group feeding a mixture of wet and dry food. Despite the potential impact of CKD on energy levels, most dogs in the CKD group (77.9%) were reported as being “high energy.”

#### 3.1.2 Demographic characteristics of dogs with CKD

**Figure 3:**
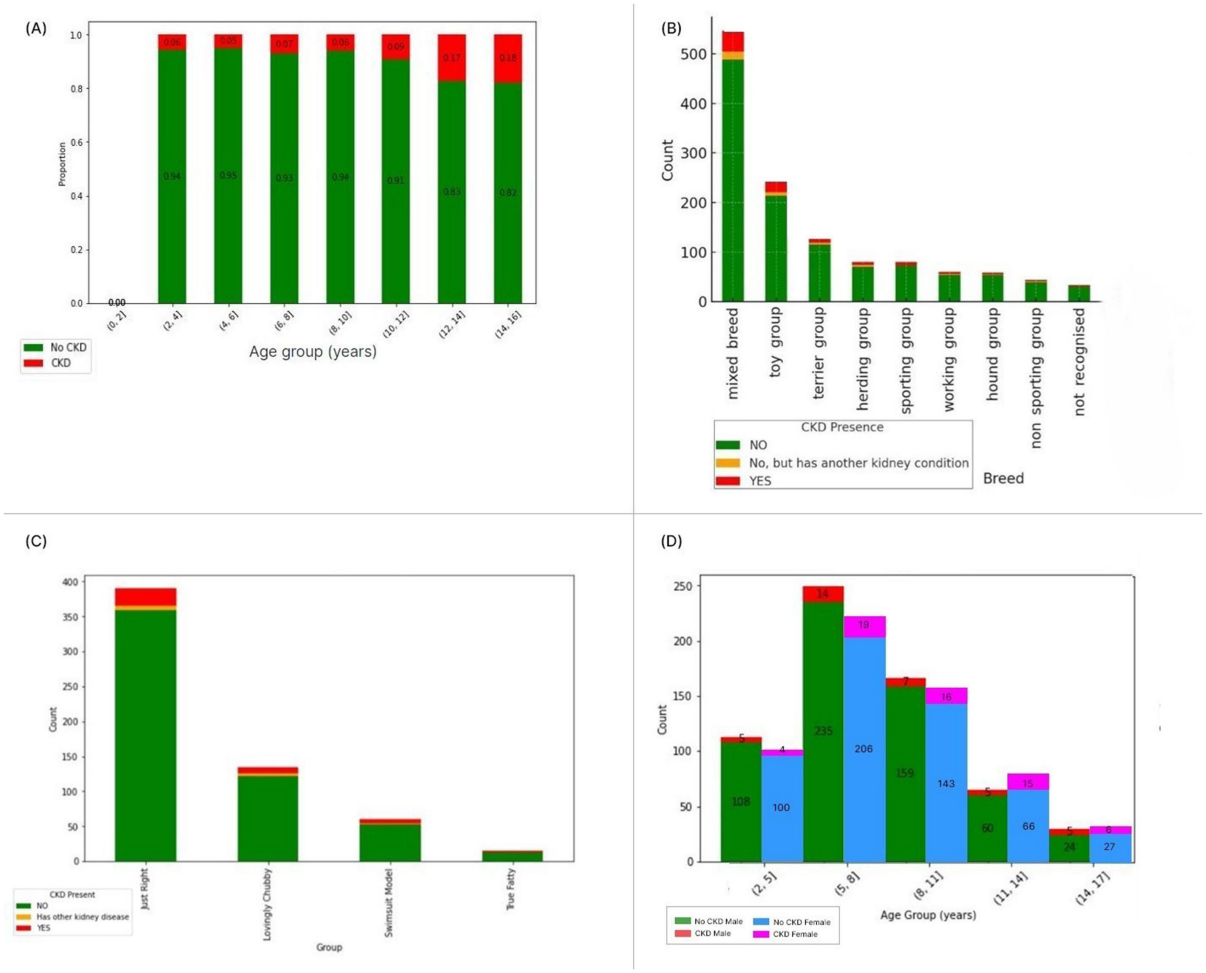
Demographic distribution of dogs with Chronic Kidney Disease (CKD) (red), healthy dogs (green) and dogs with other kidney conditions (orange) as **(A)** proportion of dogs in the survey with CKD compared to healthy dogs, **(B)** CKD prevalence across American Kennel Club (AKC) breed groups expressed as total count, **(C)** distribution by weight profile category as used in the survey from Good Boy Studios Pet Parade mobile application, **(D)** CKD prevalence by age group, comparing male (green for healthy male and red for CKD male dogs) and female dogs (blue for healthy females and pink for females with CKD).

The study population demographics are summarized in Figure 3. The sample included dogs across seven age groups weight profiles were categorized as: Swimsuit Model, Just Right and Overweight (a group that combined the Lovingly Chubby and True Fatty categories from Pet Parade as shown in Figure 3C), Supplementary Figure S2 (23).

#### 3.1.3 Pet owner observations of CKD clinical signs

The survey probed pet owners on observed clinical signs in their dog. We report on the raw clinical sign count in the cohort, and those judged by the pet owner to be the most troubling.

**Figure 4:**
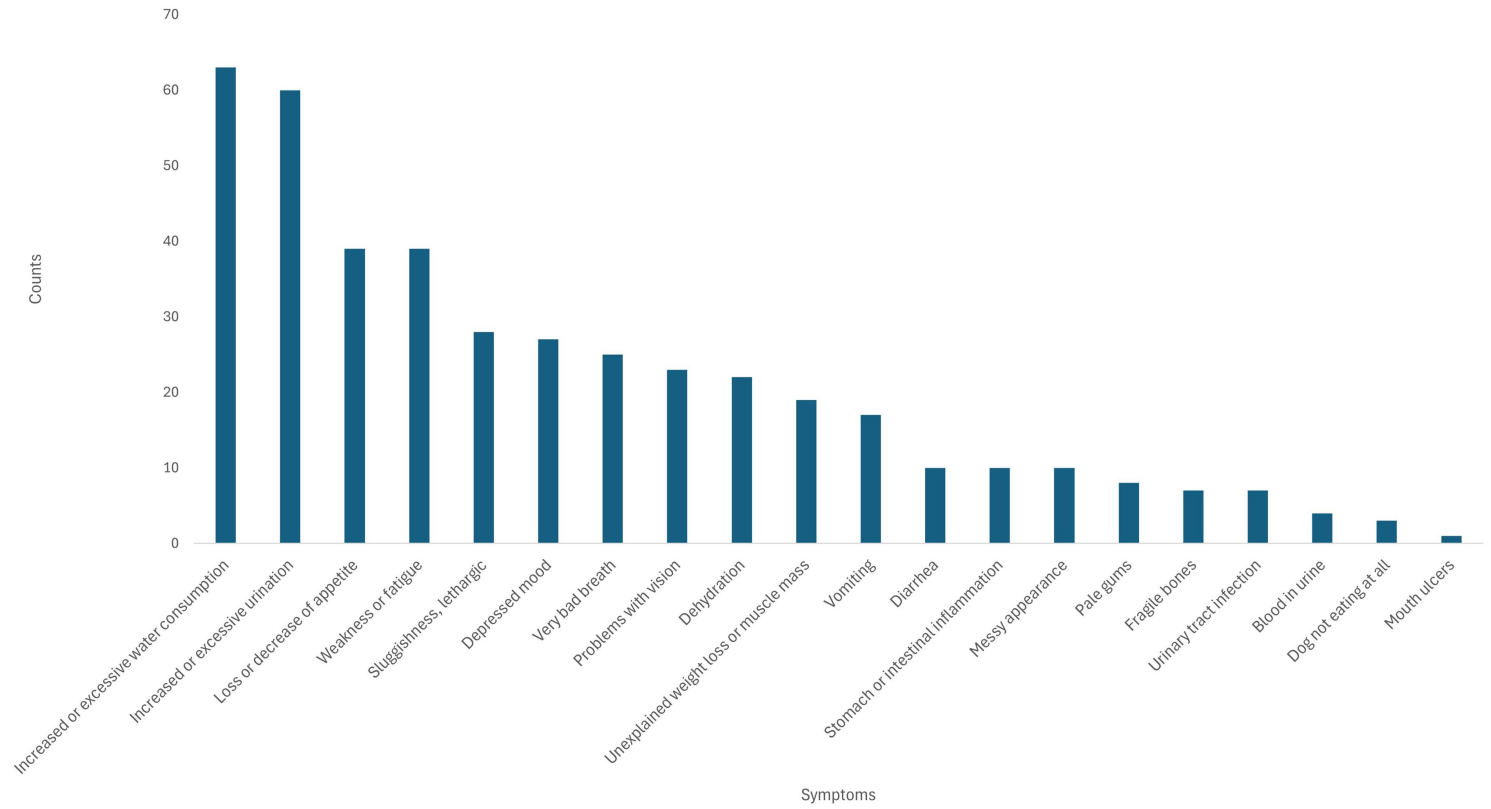
Clinical sign (referred to as “symptom” in the survey) count in dogs with chronic kidney disease (CKD) as observed by pet owners who completed the survey.

The most frequently reported clinical signs were “Increased thirst or excessive water consumption” (14.9%, *n* = 63) and “Increased or excessive urination” (14.2%, *n* = 60). These clinical signs were reported considerably more than the next most frequently mentioned clinical signs “Loss or decrease of appetite” and “Weakness or fatigue” (9.2%, *n* = 39 for both), see Figure 4. This may be attributed to fewer dogs reported in end stage kidney disease compared to those with less advanced disease progression.

**Figure 5:**
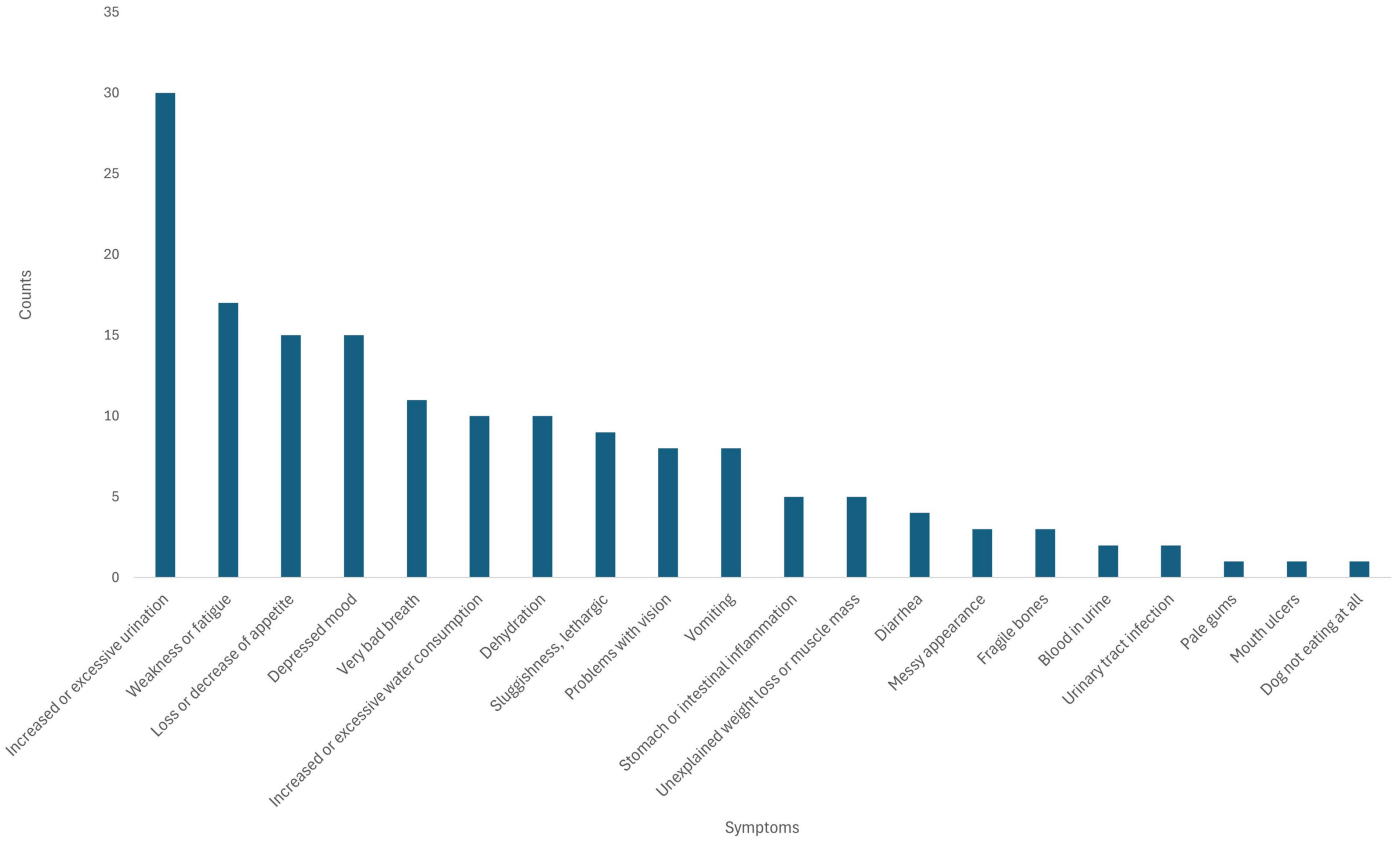
Most troubling clinical signs (referred to as “symptoms” in the survey) observed in dogs with chronic kidney disease (CKD) to pet owners. The numbers above the bars represent the count of most troubling clinical signs/raw count for the same clinical sign.

Figure 5 demonstrates which of the clinical signs of CKD observed by surveyed pet owners in their dogs they considered to be the most troubling to pet owners. “Increased or excessive urination” features prominently (*n* = 30/60 cases as reported in Figure 4), representing the highest scoring category for pet owner designated most problematic clinical sign, as well as being the most frequent clinical sign in the raw clinical sign count data. With half of those dog owners claiming this was the biggest issue they observed of those exhibiting this clinical sign. However, despite “Increased or excessive water consumption” being the second most common clinical sign in the survey group (Figure 4), it ranked as sixth most concerning to pet owners of CKD dogs (Figure 5). It therefore appears that pet owners do not appear to be as troubled by this clinical sign as its prevalence in this cohort might indicate.

The ANOVA correlation analysis of clinical signs found that the correlations were generally weak across the set of clinical signs. The most correlated pairs of clinical signs shown were (not eating AND mouth ulcers), (pale gums AND blood in urine), and (excessive/increase drinking AND Increased/ excessive urination). All other correlations were < 0.5 and not deemed meaningful or statistically significant. For example, only one dog presented with mouth ulcers, and this was associated with 3 cases of not eating; only 4 cases of blood in urine and 8 cases of pale gums. Only the correlation (of 0.66) of water consumption (63 cases) and excess urination (60 cases) was considered significant. This also associates with pet owner concerns around excess urination and drinking seen in both SML and survey data.

#### 3.1.4 Statistical analysis of the survey data

Age demonstrated a significant association with CKD diagnosis (*p* = 0.001) using a Chi-squared test. While we found a significant relationship between age and CKD status, the presence of CKD in dogs across all age groups suggests multiple factors may be associated with the condition. Logistic regression analysis revealed that the odds of CKD diagnosis increased by approximately 0.1017 for each year of age (*p* < 0.001), holding all other variables constant (21).

Gender showed a significant association with CKD diagnosis, with female dogs showing higher CKD prevalence even after accounting for their greater longevity compared to males (23, 24). Further details on logistic regression and statistical analysis can be found in section 1.5 of the Supplementary materials.

#### 3.1.5 Accessing clinical practice data for research

Where pet owners are engaged in the management of CKD in their dog, there may be opportunities for veterinarians to work with pet owners to gather additional data on the disease pathway for CKD. To answer this question, pet owners completing the survey were asked whether they would be prepared to use a home blood sampling kit (51.5% said yes), if they would be prepared to take their dog to their veterinarian for a prospective blood sample to be taken (54.6% said yes) or if the pet owner would be prepared to share routine blood test and urinalysis information from their veterinarian directly (62.9% said yes). The results are shown in Supplementary Figure S3. Pet owners were also asked if they would be willing to upload digital or paper copies of their dog’s medical records. Most pet owners (84.5%) reported not having immediate access to their dog’s medical records, either in paper or digital form. While these records are legally accessible to pet owners upon request from their veterinarian, the additional step of requesting and obtaining the records could potentially impact participation rates in studies requiring this information. Further detail appears in Supplementary Figure S4.

### 3.2 Results of social media analysis

#### 3.2.1 Overview of social media data collected

The SML study was conducted to complement the results of the survey data and further enhance insights into pet owner understanding and perceptions of CKD. Initial scoping searches were conducted to ensure that there was useful content available across all the social media platforms being scraped. We found that X generated an average of 79 posts per month on CKD-related content while Reddit, blogs and forums averaged 927 posts per month and Facebook 1,100 posts per month on content that appeared to be connected with canine CKD.

The five year (5Y) data extraction from X in isolation yielded a total of 25,374 posts. This was reduced to 16,374 valid posts following data pre-processing removing duplicates, re-tweets, special characters (e.g., emojis) and advertising as described previously. After a relevancy filter was applied, there were 14,069 posts remaining for analysis.

The one year (1Y) data scrape of blogs, forums and Reddit returned 3,039 posts initially. Data pre-processing similar to that used on X data reduced this to 1,501 and the relevancy filtering reduced this further to 616 posts. The majority of these filtered posts originated from Reddit. Few blog posts were collected using data scraping.

The broadest data scrape obtained was for a combination of social media platforms: X, blogs, forums, Reddit, Facebook and Pinterest. The data was collected for a 30 day period (1M dataset), yielding 1,521 posts at the initial data extraction stage: 1,048 after data pre-processing and 586 following relevancy filtering. X, Reddit and Facebook posts dominated the dataset although some relevant posts were retrieved from Pinterest.

#### 3.2.2 Sentiment analysis of social media discussions on CKD

**Figure 6:**
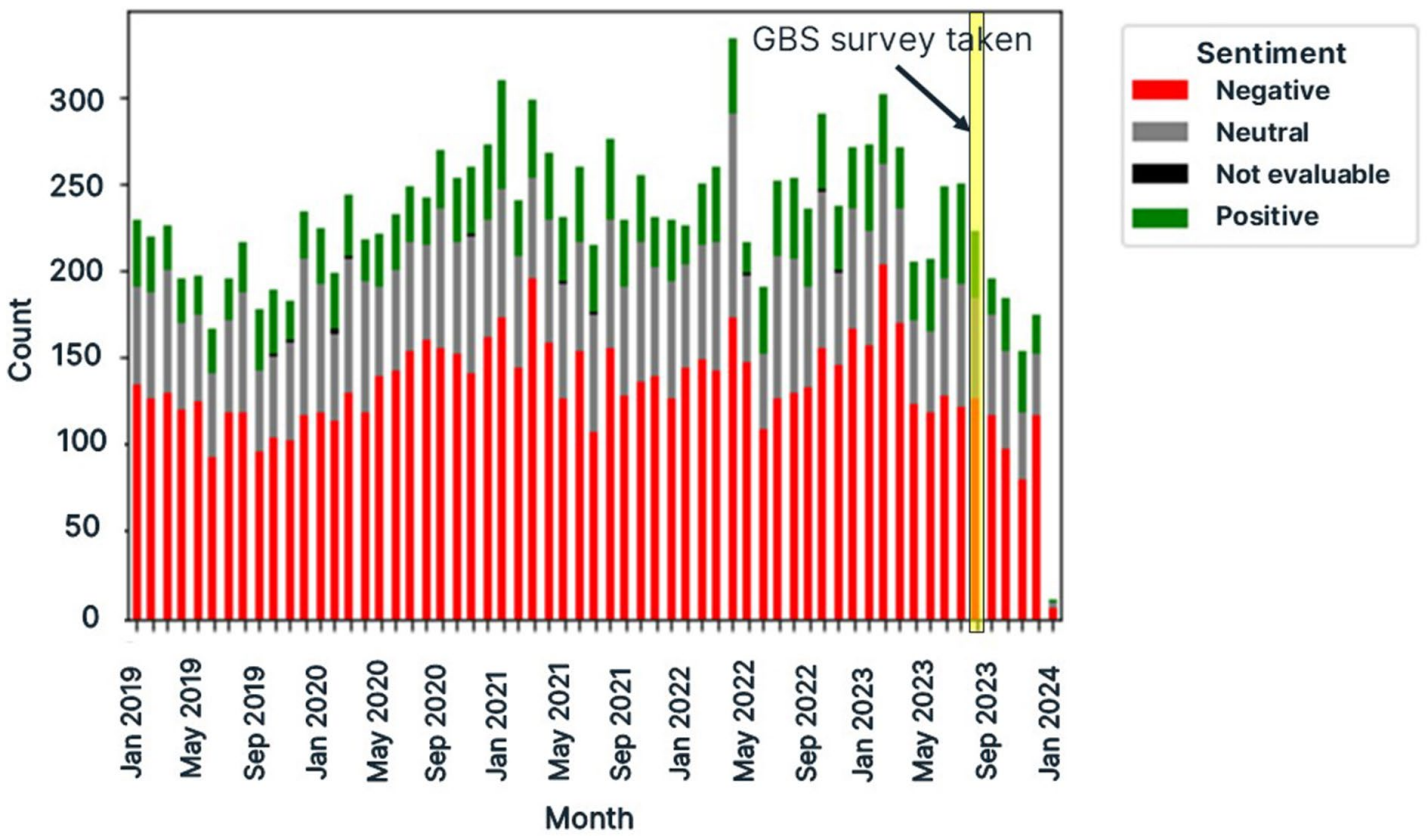
Monthly counts of posts relevant to dogs with chronic kidney disease (CKD) as retrieved from X/Twitter in a five year period from 1st January 2019 to 1st January 2024 using Pulsar Platform™.

After filtering the five year data from X for relevancy, monthly counts of posts by sentiment (positive, negative and neutral) demonstrated that sentiment of the CKD-related posts was largely negative as shown in Figure 6. This aligns with our previous SML sentiment analysis findings (14), which also indicate that SML users are more motivated to post on negative topics than positive ones (14).

**Figure 7:**
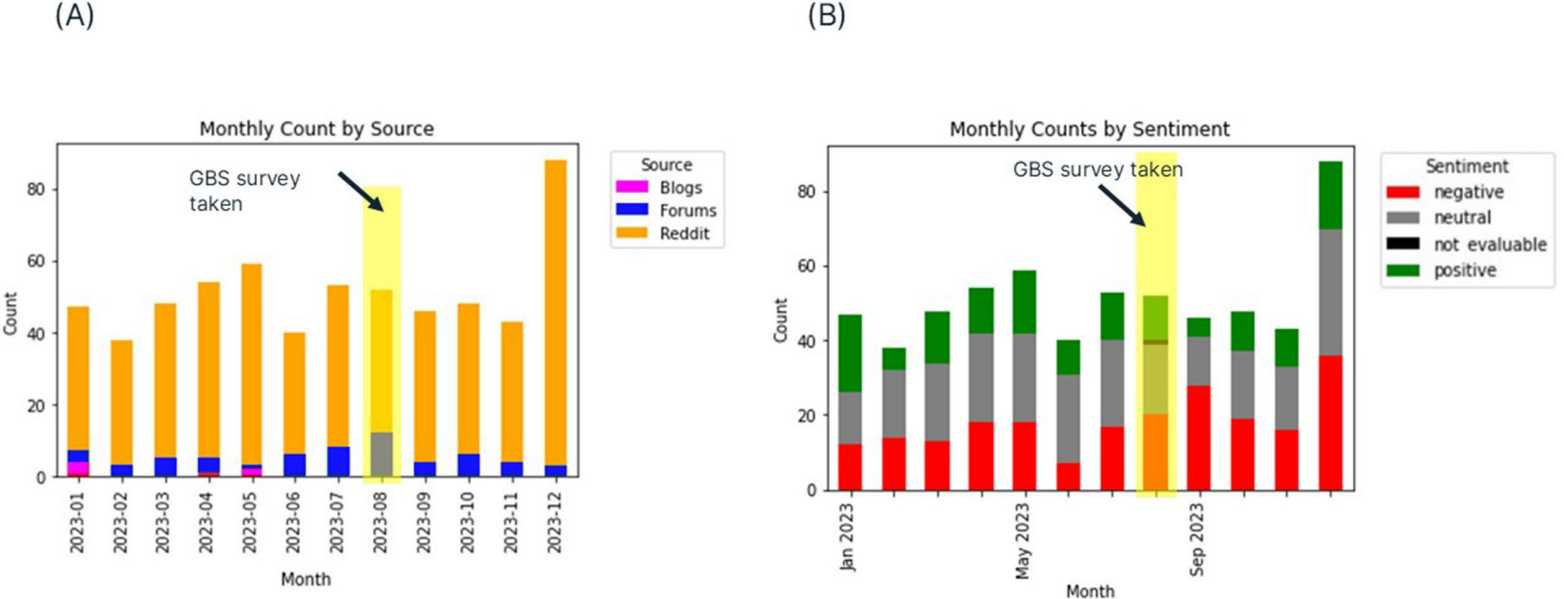
Monthly counts of posts relevant to dogs with chronic kidney disease (CKD) as retrieved from blogs, forums and Reddit in a one year period from 1st January 2023 to 31st December 2023 using Pulsar Platform™ by **(A)** source and **(B)** sentiment. This demonstrates that the timing of the survey was within a typical period of typical content generation on social media with no major biasing events in evidence.

The results from one year data from blogs, forums and Reddit were visualized by sentiment and by source as shown in Figure 7. The timing of the survey demonstrates this occurred over a typical mix of positive and negative sentiment on social media, devoid of any impactful major events that may have biased responses. Unlike X posts, which had predominantly negative sentiment, we observed the sentiment distribution for posts from Reddit, blogs and forums was more variable month on month. This may be evidence of platform-dependent user behavior.

Sentiment for the one month combined sources dataset varied day-by-day with negative sentiment being recorded at high levels. Sentiment data was not available for two days in February 2024.

#### 3.2.3 Social media reports of CKD clinical signs

It was found that the three SML datasets were, overall, relatively consistent, apart from over-representation of “loss or decrease of appetite” being more highly reported on X. The most prevalent clinical sign reported across all SM platforms was “Unexplained weight loss or muscle mass.”

### 3.3 Comparison of survey and social media insights

#### 3.3.1 Alignment of clinical sign reporting

Although SML and survey data can independently investigate similar topics and themes, the results produced provide distinct yet complementary insights due to their different sampling methodologies. Survey data offers structured responses to specific questions, while social media captures spontaneous, unprompted discussion on lived experiences. Where surveys might be limited by question design and participant self-selection, social media content reflects natural discussions but may be biased toward those who choose to share publicly. To this end, we considered the most frequently mentioned clinical signs seen in both survey and SML data.

The clinical signs most prevalent in the survey were “Increased thirst or excessive water consumption” and “Increased or excessive urination” (Figures 4, 5). This compares with the survey respondents who reported that the clinical sign they found the most troubling for their dog was “Increased or excessive urination” while “Increased or excessive water consumption” appeared to be less troubling. Further statistical analysis of the survey data indicated that the most troubling clinical signs pet owners themselves were: “Depressed mood,” “Increased or excessive urination” and “Stomach or intestinal inflammation.”

**Figure 8:**
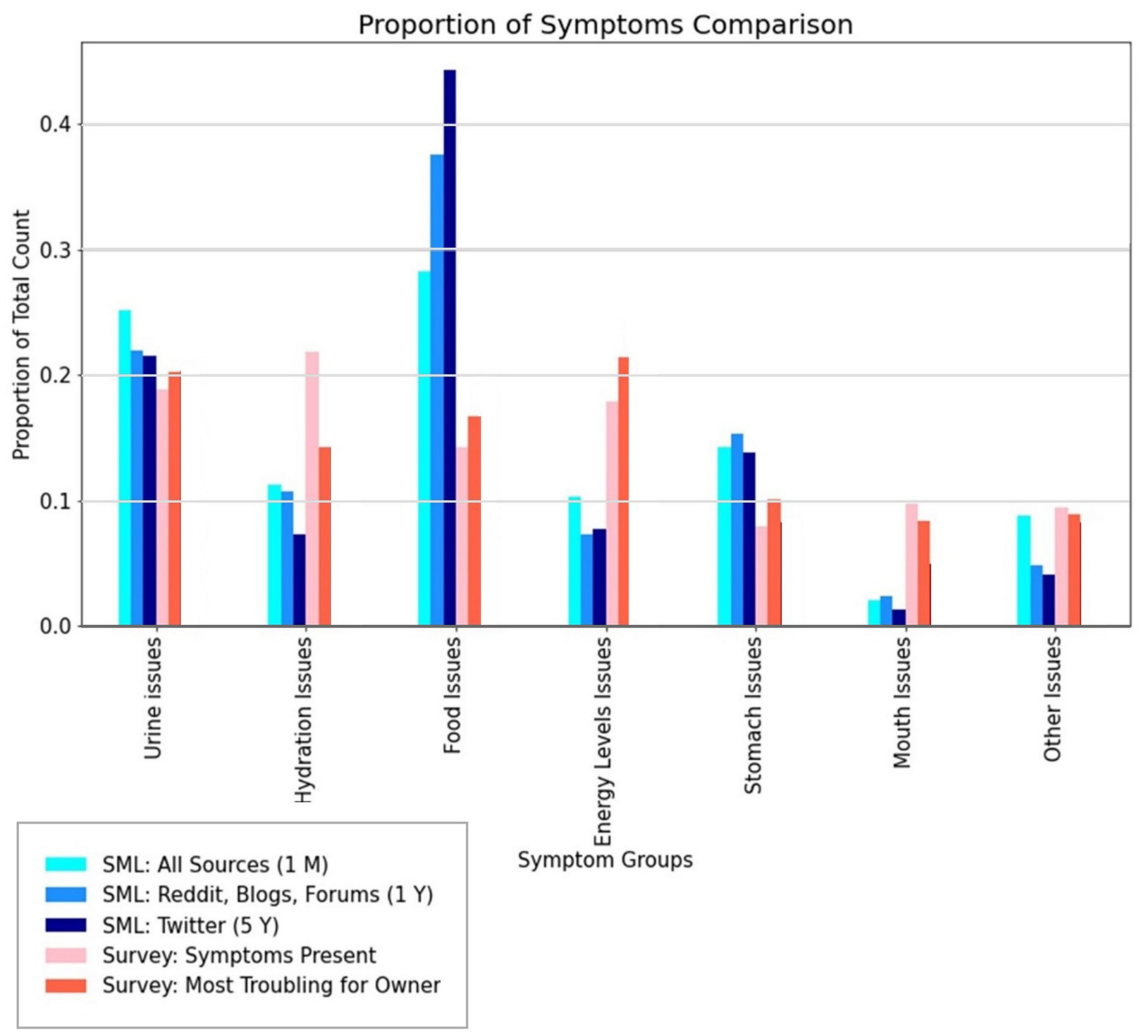
Comparison of social media listening data regarding grouped clinical signs (referred to as symptoms to match the language used in the survey, see Table 1) reported by pet owners taken from all selected sources (X/Twitter, Reddit, blogs, forums, Facebook and Pinterest) from all three searches conducted using Pulsar Platform™ with survey questions on clinical signs (symptoms) present in dogs with chronic kidney disease (CKD) and whether those clinical signs were troubling for the pet owner.

We compared the SML data from all scraped social media sources collected during a 30 day (1M dataset) period to the survey results taken from questions relating to clinical signs. This dataset was considered the most unbiased of the SML datasets because it was scraped from multiple social media sources. Figure 8 presents a comparison between clinical signs in SML data from all selected sources and those reported in the dogs described in the survey, demonstrating broad agreement with similar proportions across both data sources, particularly in recognizing urine issues in CKD (20). Both SML and survey data identify similar sets of concerning clinical signs and are qualitatively in agreement (within a factor ~ 2) for other recognized clinical signs across both data sources. While urine and energy issues rank as similarly most important for the survey results, followed closely by food and hydration issues, we see that the SML data ranks food followed by urine issues as of primary and secondary importance followed by stomach and hydration issues. Thus, while the broad ordering of importance by propensity is related there are key differences. This may be explained simply by prompting clinical sign questions as compared to unprompted social media narrative.

#### 3.3.2 Novel insights from social media: pain in CKD discussed by pet owners

Although not explicitly investigated within the survey, pain emerged as a significant concerning clinical sign in social media data (25). The absence of pain-related questions in our survey highlights how social media analysis can produce data-driven insights that might otherwise be overlooked in structured questionnaires, demonstrating the complementary value of these different data collection approaches.

The three clinical signs datasets (1. Reddit/blogs/forums, 2. X, and 3. the 30 day (1M) “all sources” dataset) were analyzed for mentions of pain in relation to canine CKD. Posts containing “pain” were filtered and manually reviewed for relevance in the smaller datasets, while an LLM was used to classify pain-related posts in the larger X dataset. After filtering and review, the datasets yielded 17, 30, and 129 pain-relevant posts, respectively. Finally, the combined pain-related posts were analyzed by an LLM to extract key topics. The analysis revealed that the top five topics associated with pain were: CKD clinical signs and progression, end-of-life decisions, pet owner grief, dog pain and suffering, and quality of life considerations. A further 10 pain-related topics were found, and these are presented in the Supplementary materials.

## 4 Discussion

Canine CKD is a progressive and ultimately fatal condition. This work has shown that CKD prompts pet owners to seek support and information on social media platforms. Our research employed both traditional surveys and innovative SML techniques to explore pet owner experience of CKD in dogs, addressing our primary research question of what insights can be gained by comparing pet owner survey responses with social media discussions. While each method had its limitations, together they offered a more comprehensive understanding of how pet owners experience and manage their dog’s condition. The complementary nature of survey and SML methods was particularly evident in their temporal perspectives. Survey data provided a structured snapshot of post-diagnosis experiences, while social media has the potential to capture a variety of episodes in the patient journey from initial clinical signs through diagnosis and management. This temporal range can help to build a more complete picture of the CKD journey from the pet owner’s perspective.

Despite the initial broad response (1,633 participants), only 132 responses provided data specific and relevant to dogs with CKD, or another kidney or renal condition. This demonstrates the challenge of recruiting specific disease populations and pet owners’ willingness to participate. Statistical analysis revealed significant correlations with age (*p* < 0.001) and female gender (*p* = 0.006), with females showing higher CKD prevalence, despite accounting for their greater longevity, and each year of age increasing CKD correlation by approximately 0.1017, reflecting the progressive nature of CKD (2). While no significant correlations emerged between CKD presence and factors like breed or weight, contradicting some breed-specific prevalence studies (2), this aligned with VetCompass™ which reported similar demographic trends in CKD prevalence (4). However, the relatively small sample size and subjective nature of some measures in the survey limit the generalizability of these findings. Moreover, as female dogs tend to live longer than their male counterparts, this gender divide may influence some of the findings (23, 24). See Supplementary Table S2 for a summary of the survey data demographics (pet gender, adoption, food type, animal size, fur type, energy level and country).

Leveraging the zero-shot capabilities of LLMs for classification (filtering) of SML data proved to be an efficient alternative to traditional supervised machine learning approaches. While training a machine learning model classifier from scratch would have required extensive and laborious expert data annotation and model training, our innovative LLM-based approach demonstrated greater flexibility and scalability in analyzing unstructured data. The ability to generalize to new classification tasks without requiring extensive expert labeled datasets represents a significant advantage (18, 19).

SML data provided a broad spectrum of unstructured public discourse, offering real-time insights into how pet owners perceive and manage CKD. The three SML datasets were relatively consistent, apart from over-representation of “loss of appetite” on X. It is unclear why this phenomenon was observed, other than conjecturing that the variations between social media platforms may reflect differences in user demographics and potential biases in how they observe and report pet health. Further investigation of these platform-specific differences could inform targeted strategies for research recruitment and educational initiatives. Unsurprisingly, the analysis of sentiment and content via SML revealed a predominance of negative sentiments, particularly on platforms like Reddit and X, echoing findings by Cherry et al. (14) on the public’s proactive engagement in discussing troubling clinical signs for an incurable disease. This aspect of SML highlights both its reach and the inherent biases of user-generated content, as it captures a more engaged, often distressed user base, which may not represent the general pet owner population.

The clinical sign analysis from the survey correlated well with SML data, where increased thirst and urination were frequently discussed clinical signs, highlighting their impact on quality of life and care complexity. While these clinical signs were frequently reported in both datasets, social media discussions highlighted additional concerns not captured in our structured survey. It is interesting to observe that “unexplained weight loss or muscle mass” was the most prevalent clinical sign reported across social media platforms, whereas this ranked lower in survey responses. This may be due to the nature of scraping social media where pet owners without a confirmed diagnosis are searching for information online and reporting these as early clinical signs, whereas our survey data only represented those dogs post diagnosis, and thus further advanced in the disease pathway. The survey data was more effective than the SML data in understanding how CKD clinical signs appear in combination, as SML posts typically mentioned relatively few clinical signs together. However, when comparing SML data with survey responses, the strongest correlation was found with clinical signs that owners identified as “most troubling.” This suggests that social media analysis may be particularly effective at capturing owner concerns about severe or distressing clinical signs both pre- and post-diagnosis.

These findings have important implications for both veterinary practice and future research methodology. The combination of structured surveys and social media analysis provides a richer understanding of pet owner experiences. These underscore the importance of understanding pet owner-reported clinical signs and the emotional burdens carried by pet owners. This understanding is vital for veterinarians and researchers to develop more targeted interventions such as quality of life measures and communication strategies that address both the clinical aspects of CKD and the quality-of-life considerations for both pets and their owners. Future studies might benefit from using initial social media analysis to identify key themes for subsequent survey development, creating an iterative approach to understanding pet owner perspectives on chronic conditions. Our study also demonstrated pet owner willingness to contribute to CKD research, suggesting potential for ongoing data collection efforts.

The SML approach complements traditional research methods by identifying topics that may not have been initially considered in structured survey designs. This demonstrates how SML can be used to understand free-form conversations around pet owners and observations relating to a particular disease type thereby identifying important topics that might otherwise be overlooked. The unstructured nature of social media conversations reveals insights that may then be used to inform the content of follow-up survey design and focus group questions. However, the proviso on using such data is that careful filtering is required to ensure that only pertinent information is used in any subsequent analyses, as phrases may have multiple meanings across different topics.

The analysis of SML posts reveals that pain is a significant concern for pet owners dealing with canine CKD, particularly when considering end-of-life care. While pain may not always be directly observed in dogs with CKD, or pain may be managed with medication, pet owner anxiety surrounding potential pain and suffering is a recurring theme in online discussions. According to Dadousis et al. (25), although pain has an estimated prevalence of 50–70% in human CKD patients, it remains potentially undertreated and may not receive sufficient consideration in veterinary medicine. The decision to euthanize is often framed as a humane choice to prevent or end pain, though it frequently leads to emotional distress among pet owners. These findings underscore the complex interplay between observed clinical signs, perceived suffering, and the emotional challenges faced by pet owners managing canine CKD. Due to the challenges in reliably detecting and assessing pain in companion animals, findings from human studies may help inform more targeted pain assessment and management approaches in veterinary medicine. The relative lack of pain-related discussions in veterinary literature compared to human medical literature suggests this may be an important area for future research.

### 4.1 Limitations

The described survey was geographically restricted to the US and UK, with most posts originating from the US, and limited to English speakers during a specific time frame (August 2023 to January 2024). SML data was scraped for 30 days on social media (blogs, forums, Facebook, Pinterest, Reddit and X), one year on Reddit, blogs and forums; and 5 years on X with the search expression set to English Language posts only. In both cases, other factors or insights, whether regional or otherwise, may have been surfaced if additional non-Anglophone posts were considered.

Notably, only 132 out of 1,633 survey respondents were relevant to the CKD dog study, highlighting the challenge of obtaining a focused sample. Additionally, the survey revealed that when owners identified their most troubling clinical signs, some clinical signs were not mentioned as present in the previous question. This suggests that future surveys could be optimized by only showing clinical signs checked in the previous question, thereby enhancing data quality and logical consistency.

Within the SML data it is possible that multiple unique posts may refer to the same animal, identifying such cases with certainty would be challenging due to the nature of social media data extraction, where posts are analyzed episodically and contextual connections between posts are not preserved. It may be useful to develop further methods to filter out such posts.

### 4.2 Future work

This work has highlighted opportunities for further insights that can be gained from both survey data and SML and judicious use of both in combination. Further surveys and SML investigation could be used to address pet owner insights and target any differences in insight, experience and understanding between pet owners and veterinarians prescribing care for CKD dogs.

Further understanding of observable clinical signs and correlating these with disease severity could also be used in the development of novel quality of life measures and potentially as predictors for disease progression.

Although we found no statistical significance in investigating CKD presence with breed, this was limited by our dataset size. A larger dataset might make it possible to prove/disprove this hypothesis with greater statistical power.

## 5 Conclusion

This paper has examined CKD understanding in pet owners using both survey and SML methods, addressing our primary research question regarding insights gained from comparing pet owner survey responses with social media discussions. We found there were many common concerns (e.g., food issues including loss of appetite) and a set of common clinical signs reported (energy levels, food issues/weight loss etc.), albeit in differing proportions across the different data sources. This variation may be explained by the fact that survey data required dogs with known CKD issues, whereas our SML data captured data pertaining to canine CKD as part of the online discourse. This means that pet owners posting online may or may not have a confirmed diagnosis and may therefore be at an earlier point in terms of disease progression compared to those in our survey. The survey respondents demonstrated their willingness to contribute data to future research studies with 63% willing to share routine blood and urinalysis test results from their veterinarian.

Our statistical analysis of survey data demonstrated that there was a significantly higher prevalence of CKD for female dogs as compared to male dogs, even when accounting for the longer lifespans of females compared to males. We also found that age showed a significant association with CKD diagnosis, although the presence of CKD across all age groups suggests multiple contributing factors. While we found no significant associations between CKD presence and breed group or (semi-quantitative) weight category, this may be due to low statistical power in our sample.

By comparing traditional survey methodology with the innovative approach of SML and advanced data analysis techniques, we have demonstrated similarities in these datasets but also key differences (e.g., pain emerged as a significant novel theme in the social media data but no pain-related questions were presented in the survey as this is not typically seen in CKD). These differences reflect the different populations sampled and the manner in which such dog owners freely post and/or respond to structured survey questions. The complementary nature of these approaches enhances our understanding of how pet owners experience and manage canine CKD.

## Data Availability

The data supporting the conclusions of this article will be made available by the authors, without undue reservation.
