# Supplementary material for: Gaining insights into pet owner understanding/lived experience of canine chronic kidney disease using survey and social media data
Source: Front Vet Sci. 2025 May 7;12:1506272. doi: 10.3389/fvets.2025.1506272 (PMC12094028; doi:10.3389/fvets.2025.1506272)
Supplement: Supplementary file 1 [file Data_Sheet_1.docx]

Supplementary Material

Gaining insights into pet owner understanding/lived experience of Canine Chronic Kidney Disease using survey and social media data

Georgina Tarrant^1†*^and Luke Boyden^1,2†^, Taranpreet Rai^1,2^, Andrea Wright^3^, Alasdair J.C. Cook^1^, Kevin Wells^1,2^

^1^Surrey DataHub, vHive, School of Veterinary Medicine, University of Surrey, Guildford, United Kingdom

^2^Centre for Vision Speech and Signal Processing, University of Surrey, Guildford, United Kingdom

^3^Outcomes Research, Zoetis, Loughlinstown, Dublin, Ireland

*** Correspondence:**Georgina Tarrant
georgina.tarrant@surrey.ac.uk

# Supplementary Data

## Various forms of pain as discussed by pet owners when referring to dogs with CKD

The three SML datasets used for the comparison of symptoms were analyzed for mentions of pain in relation to canine CKD. Posts containing "pain" were filtered and manually reviewed for relevance in the Reddit, blogs, and forums data, and for the 30-day “all sources” data. For the larger Twitter dataset, an LLM was used to classify pain-related posts.

The 'all sources' dataset had 586 posts, with 17 pain-relevant posts after filtering by “pain” (n=53) and reviewing the content for relevance to pain. The Reddit/blogs/forums dataset had 616 posts, with 30 pain-relevant posts after filtering (n=68) and manual review. The X dataset, spanning 5 years, had 14,069 posts (545 mentioning pain). A LLM classified these due to volume. The 144 classified posts were reduced to 129 after the removal of duplicates.

The social media posts about pain and CKD were combined and a LLM was prompted to extract key topics from the data. The analysis identified 15 main topics, including:

- CKD symptoms and progression
- End-of-life decisions
- Pet owner grief
- Dog pain and suffering
- Quality of life considerations
- Euthanasia experiences
- Treatment options
- Owner anxiety and guilt
- Behavioral changes in dogs
- Pain management importance

"I lost her to CKD about 6 weeks ago and while my heart feels like it's missing a really big piece, knowing she's no longer in pain eases the hurt."

## Physical pain and suffering experienced by dogs with advanced CKD

"Renal disease is a wasting disease and the calcification of her organs has begun. She is getting more and more lethargic and equally demanding.... that's kinda funny at times, typical Catahoula = hard headed."

"Worst of all, Binoy is in so much pain due to his inability to pee that his bladder burst inside. An emergency surgery, a cystotomy, had to be done immediately to ease his pain and suffering from his renal problem."

## Quality of life considerations for dogs with CKD

"We were told it's better to let our dog go sooner rather than later but she's still doing a lot of stuff that she enjoys except for maybe running which I've only seen her do once since her diagnosis. I try to observe her to make sure shes not in pain as well."

"The vet said we could probably get 3 or 4 more weeks with him but he was already in pain and it would continue to get worse as the days go on. We opted to let him go a couple of days later. Had a vet come to the house and he was still super active up til the sedated him."

## Further information about Pet Parade mobile application survey

The survey was conducted from August 2023 to January 2024 using the Pet Parade mobile application, owned by Good Boy Studios (GBS). Pet Parade is a social platform application available in the USA and UK, where pet owners share photographs and videos to participate in competitions for virtual prizes and gift cards. The platform requires users to verify their identity via email or text verification and create comprehensive pet profiles including the pet's name, gender, weight, breed, birthdate, home country and known health conditions. All profile information is self-reported by users during registration. Pet Parade was used in this study to reach English-speaking dog owners in the US and UK to investigate whether they might share veterinary medical records and participate in health-focused research. Survey participants were recruited from existing app users through in-app advertisements. When a user clicked on an advertisement to accept and complete the survey, their pet’s profile information was automatically copied over to the survey without the pet owner having to re-enter this information. Only participants who reported having a living dog diagnosed with CKD (by a veterinarian) were asked to complete the full survey, receiving a $10 Amazon voucher as an incentive. The survey asked pet owners to identify the symptoms their dog was experiencing and, from the same list of symptoms, indicate which were most troubling for the dog and the pet owner when providing care. Data from Pet Parade users with healthy dogs (without CKD) served as a control group, identified as those who answered “no” when asked if their dog had been diagnosed with CKD by a veterinarian.

## Further information on the statistical analysis

Logistic regression analysis confirmed that male dogs had significantly lower odds of CKD diagnosis compared to females when controlling for other variables (p=0.006). Separate logistic regressions by gender (Table S1) further supported this finding, indicating that females exhibited higher CKD prevalence across age groups despite accounting for their longer life expectancy.

Analysis of AKC breed groups showed no significant association with CKD diagnosis (p=0.98). Similarly, weight profile showed no significant correlation with CKD diagnosis, whether analyzing three categories excluding True Fatty (p=0.59) or combining overweight categories (p=0.58). While obesity is associated with CKD development and progression in humans (25) and being underweight affects survival rates in dogs with CKD (4,26,27), our data did not demonstrate a significant association between weight profile and CKD diagnosis in this population.

# Supplementary Figures and Tables

## Supplementary Figures

**
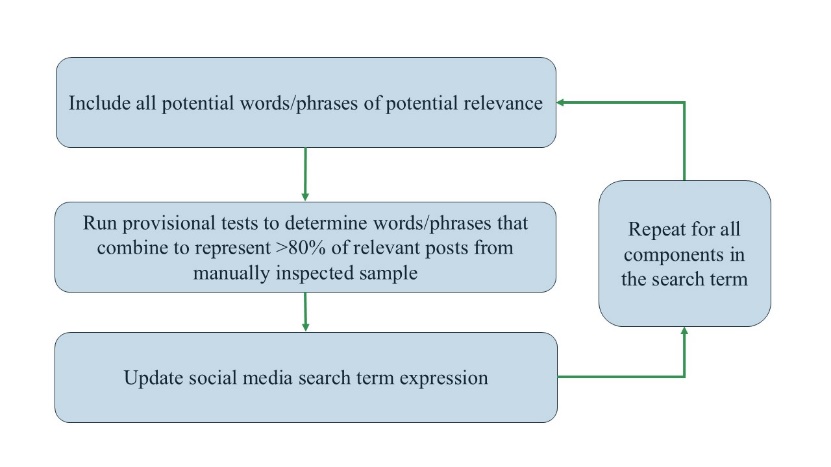
**

**Figure S1. Boolean search expression development process to ensure sensitivity and recall of relevant posts are optimized.**

**
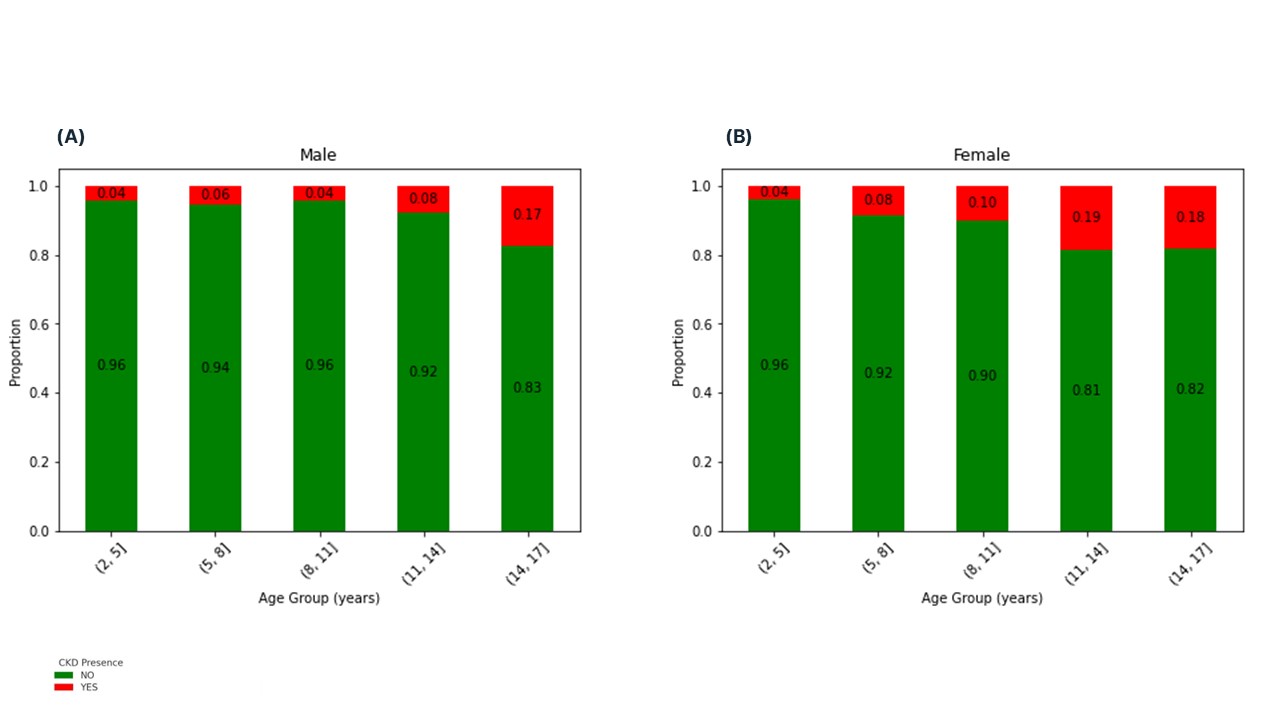
**

**Figure S2. Dogs diagnosed with chronic kidney disease (CKD) (red) and those without CKD (green) by age group for male dogs (A) and female dogs (B) expressed as proportions.**

**
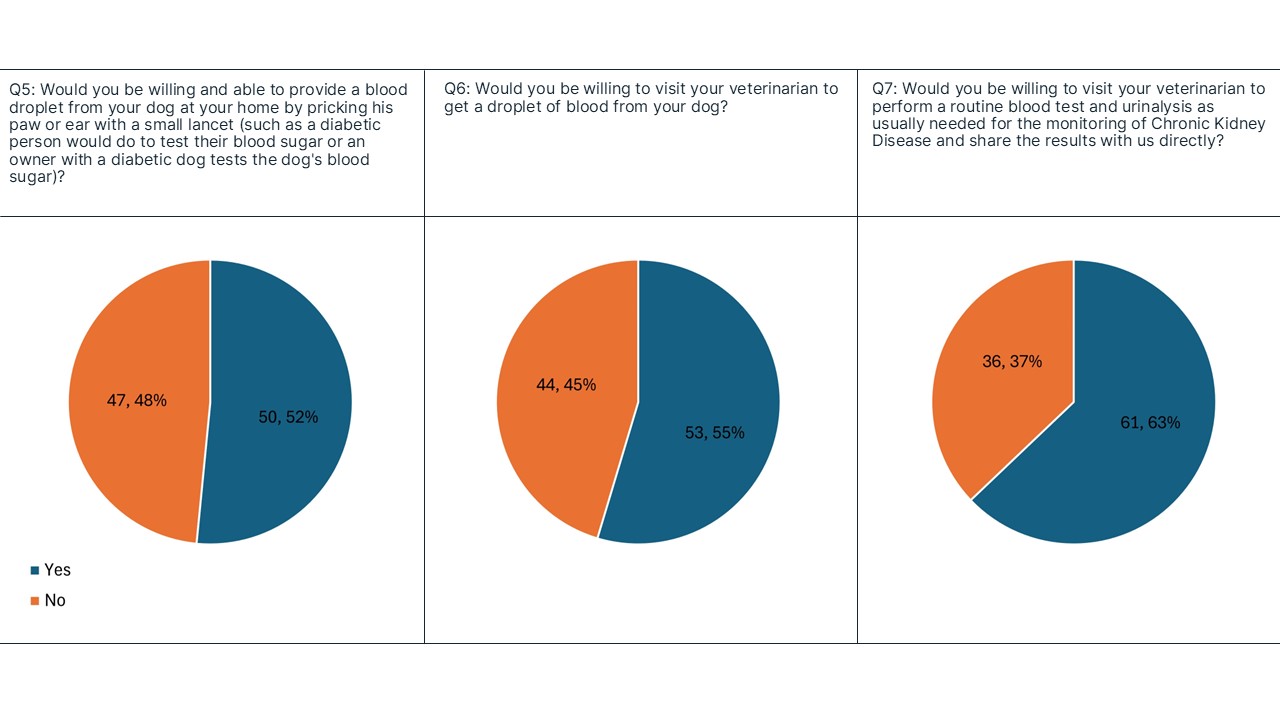
**

**Figure S3. Survey results demonstrating pet owner willingness to provide a blood sample from their dog with chronic kidney disease either at home or at the veterinary clinic.**

**
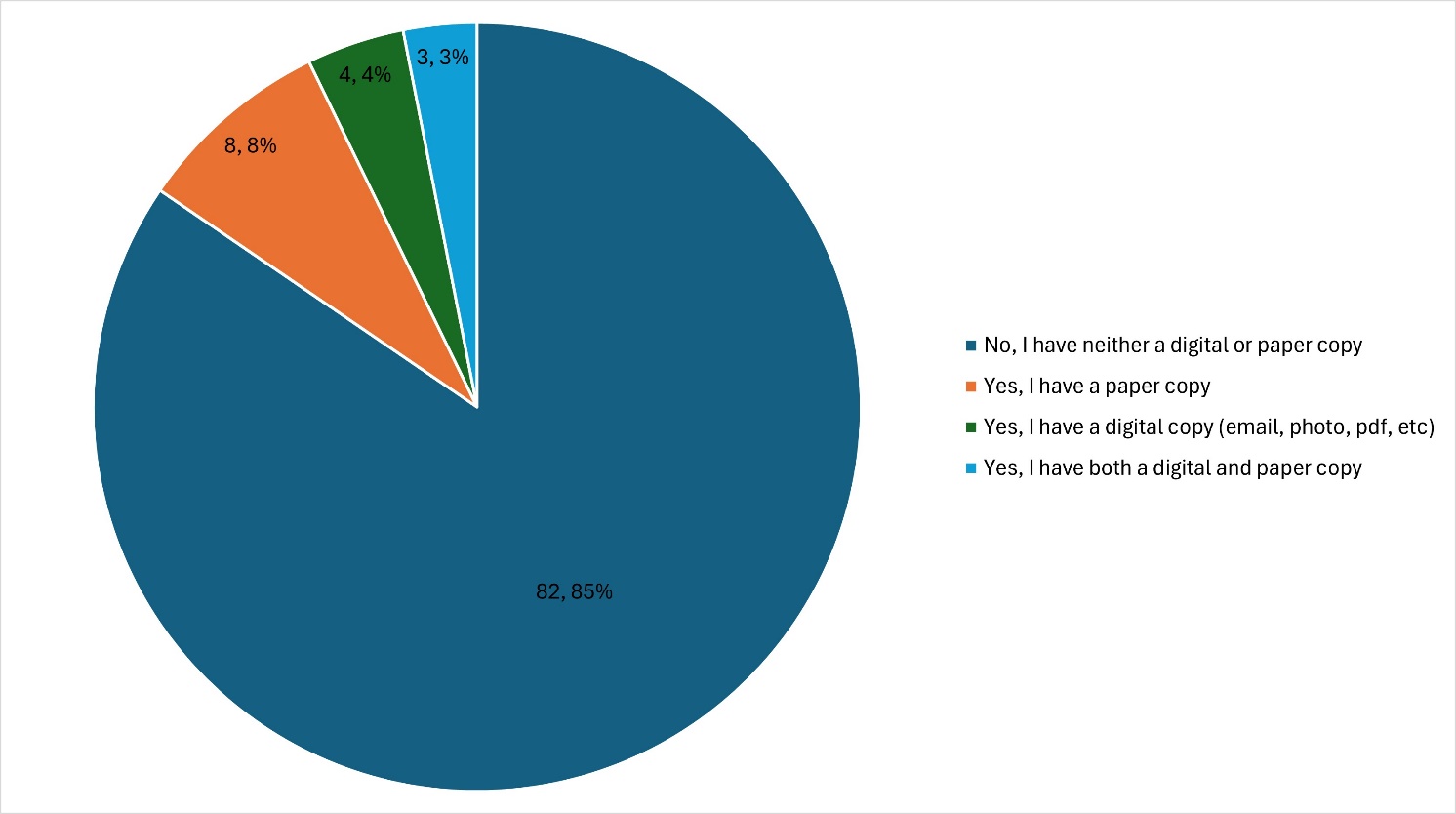
**

**Figure S4. Survey results regarding pet owner willingness to share their pets medical records.**

## Supplementary Tables

**Table S1. Summary of logistic regression analysis for age and gender.**

| **Variable** | **Coefficient (β)** | **Standard error** | **OR (e^β)** | **95% CI** | **P-value** | **Significance** |
| --- | --- | --- | --- | --- | --- | --- |
| Gender | -0.6049 | 0.220 | 0.546 | (0.355, 0.841) | 0.006 | * |
| Age | 0.1017 | 0.028 | 1.107 | (1.048, 1.170) | 0.000 | ** |
|  |  |  |  |  |  |  |

**Note: * p < 0.05, ** p < 0.001**

**Table S2 Summary statistics from the survey: pet gender, adopted from, food type, animal size, fur type, energy level, country code (GB or US).**

|  |  | **Dogs diagnosed with CKD or another kidney condition** | | | **Healthy dogs (no CKD)** | | | **All living dogs from the survey** | | |
| --- | --- | --- | --- | --- | --- | --- | --- | --- | --- | --- |
|  | **Response** | **Total no. responses (for the question)** | **Count** | **Per cent (%)** | **Total no. responses (for the question)** | **Count** | **Per cent (%)** | **Total no. responses (for the question)** | **Count** | **Per cent (%)** |
| **Pet gender** | Female | 132 | 83 | 62.9 | 1143 | 546 | 47.8 | 1275 | 629 | 49.3 |
|  | Male |  | 49 | 37.1 |  | 597 | 52.2 |  | 646 | 50.7 |
| **Adopted from** | Other | 49 | 18 | 36.7 | 544 | 117 | 21.5 | 593 | 135 | 22.8 |
|  | Friend or relative |  | 11 | 22.4 |  | 140 | 25.7 |  | 151 | 25.5 |
|  | Breeder |  | 8 | 16.3 |  | 142 | 26.1 |  | 150 | 25.3 |
|  | Animal shelter |  | 9 | 18.4 |  | 106 | 19.5 |  | 115 | 19.4 |
|  | Pet store |  | 3 | 6.1 |  | 22 | 4.0 |  | 25 | 4.2 |
|  | Adoption website or app |  | 0 | 0.0 |  | 3 | 0.6 |  | 3 | 0.5 |
|  | Other - Purchased my pet |  | 0 | 0.0 |  | 3 | 0.6 |  | 3 | 0.5 |
|  | Other - Rescued my pet |  | 0 | 0.0 |  | 11 | 2.0 |  | 11 | 1.9 |
| **Food type** | Wet and dry (both) | 16 | 9 | 56.3 | 181 | 77 | 42.5 | 197 | 86 | 43.7 |
|  | Dry |  | 6 | 37.5 |  | 96 | 53.0 |  | 102 | 51.8 |
|  | Wet |  | 1 | 6.3 |  | 8 | 4.4 |  | 9 | 4.6 |
| **Animal size** | Large | 125 | 51 | 40.8 | 1071 | 426 | 39.8 | 1196 | 477 | 39.9 |
|  | Small/Toy |  | 65 | 52.0 |  | 470 | 43.9 |  | 535 | 44.7 |
|  | Medium |  | 8 | 6.4 |  | 165 | 15.4 |  | 173 | 14.5 |
|  | Extra large |  | 1 | 0.8 |  | 10 | 0.9 |  | 11 | 0.9 |
| **Fur type** | Short | 104 | 47 | 45.2 | 924 | 462 | 50.0 | 1028 | 509 | 49.5 |
|  | Long |  | 37 | 35.6 |  | 231 | 25.0 |  | 268 | 26.1 |
|  | Medium |  | 20 | 19.2 |  | 231 | 25.0 |  | 251 | 24.4 |
| **Energy level** | High | 104 | 81 | 77.9 | 924 | 673 | 72.8 | 1028 | 754 | 73.3 |
|  | Medium |  | 19 | 18.3 |  | 194 | 21.0 |  | 213 | 20.7 |
|  | Low |  | 4 | 3.8 |  | 57 | 6.2 |  | 61 | 5.9 |
| **Country code** | US | 132 | 126 | 95.5 | 1143 | 1109 | 97.0 | 1275 | 1235 | 96.9 |
|  | GB |  | 6 | 4.5 |  | 34 | 3.0 |  | 40 | 3.1 |

## Survey Questionnaire

**Survey questionnaire:** **Chronic Kidney Disease (CKD) in Dogs**

Please help us better understand the occurrence and symptoms of dogs with Chronic Kidney Disease (CKD). Please complete all questions marked with an asterisk to receive your **$10 Amazon Gift Card**. The results of this survey will be analyzed by academic researchers whose results may be used for publication in scientific journals

Sponsored by Zoetis®

1. Is your dog ___________ currently alive?*
   1. YES
   2. NO
2. What is the current weight of __________ in ______?*
3. Has your dog __________ been diagnosed by a veterinarian with Chronic Kidney Disease (CKD) or Chronic Renal Disease? Please answer yes only if your dog has been diagnosed by a vet with these specific diseases.
   1. YES
   2. NO
   3. NO, but my dog has been diagnosed with another type of kidney or renal disease
4. What other type of kidney or renal disease has ﻿_________ ﻿been diagnosed by a veterinarian?
5. Please list out any additional diseases that ﻿___________﻿ has been diagnosed by a veterinarian.
6. Does your dog currently show any of the following symptoms? (Choose as many as you like)
   1. Increased or excessive water consumption
   2. Increased or excessive urination
   3. Dehydration
   4. Unexplained weight loss or muscle mass
   5. Loss or decrease of appetite
   6. Dog not eating at all
   7. Depressed mood
   8. Weakness or fatigue
   9. Sluggishness, lethargic
   10. Vomiting
   11. Diarrhea
   12. Very bad breath -
   13. Pale gums
   14. Stomach or Intestinal Inflammation -
   15. Mouth ulcers
   16. Problems with vision
   17. Messy appearance
   18. Fragile bones
   19. Blood in urine
   20. Urinary tract infection
   21. My dog has NONE of the symptoms above
   22. If Other add in ________
7. Of the symptoms you checked in the previous question, which are the **MOST troubling** for your **dog**? (Choose as many as you like)
   1. Increased or excessive water consumption
   2. Increased or excessive urination
   3. Dehydration
   4. Unexplained weight loss or muscle mass
   5. Loss or decrease of appetite
   6. Dog not eating at all
   7. Depressed mood
   8. Weakness or fatigue
   9. Sluggishness, lethargic
   10. Vomiting
   11. Diarrhea
   12. Very bad breath (Halitosis)
   13. Pale gums
   14. Stomach or Intestinal Inflammation (Gastroenteritis)
   15. Mouth ulcers
   16. Problems with vision
   17. Messy appearance
   18. Fragile bones
   19. Blood in urine
   20. Urinary tract infection
   21. NONE of the symptoms above are troubling for my dog
   22. Other
8. Of the symptoms you checked in the previous question, which are the **MOST troubling for you** when caring for your dog? (Choose as many as you like)
   1. Increased or excessive water consumption
   2. Increased or excessive urination
   3. Dehydration
   4. Unexplained weight loss or muscle mass
   5. Loss or decrease of appetite
   6. Dog not eating at all
   7. Depressed mood
   8. Weakness or fatigue
   9. Sluggishness, lethargic
   10. Vomiting
   11. Diarrhea
   12. Very bad breath
   13. Pale gums
   14. Stomach or Intestinal Inflammation
   15. Mouth ulcers
   16. Problems with vision
   17. Messy appearance
   18. Fragile bones
   19. Blood in urine
   20. Urinary tract infection
   21. NONE of the symptoms above are troubling for me
   22. Other
9. Would you be willing and able to provide a blood droplet from your dog at your home by pricking his paw or ear with a small lancet(sharp) such as a diabetic person would do to test their blood sugar or an owner with a diabetic dog tests the dogs blood sugar? In this example (A blood sample kit would be mailed to your home and provided free of charge.
   1. YES
   2. NO if no why?
10. Would you be willing to visit your veterinarian to get a droplet of blood from your dog? (A blood sample kit would be mailed to your home and provided free of charge. You would need to schedule an appointment with your vet and take the blood sample to your vet for your vet to collect the blood sample. In this example you would be reimbursed for the cost of the vet visit.)
    1. YES
    2. NO if no why?
11. Would you be willing to visit your veterinarian to perform a routine blood test and urinalysis as usually needed for the monitoring of Chronic Kidney Disease and share the results with us directly? (In this example you would be reimbursed for the cost of the vet visit and provided with instructions for you to share with your veterinarian).
    1. YES
    2. NO if no why
12. Do you have a digital and/or paper copy of your dog's medical records or receipts, or instructions from your most recent Vet visit in which your dog was treated for or diagnosed with Chronic Kidney Disease or Chronic Renal Disease?*
    1. Yes, I have a digital copy (email, photo, pdf, etc)
    2. No, I have a paper copy
    3. Yes, I have both a digital(email) and paper copy
    4. No, I have neither a digital or paper copy
13. Please upload medical records, receipts, and/or instructions from your most recent vet visit in which your dog was diagnosed or treated for Chronic Kidney Disease (1 of 5). Upload a single document of any format below. You can upload additional pages/documents in the next question.
14. Please upload medical records, receipts, and/or instructions from your most recent vet visit in which your dog was diagnosed or treated for Chronic Kidney Disease (2 of 5). Upload a single document of any format below. You can upload additional pages/documents in the next question. Skip if finished.
15. Please upload medical records, receipts, and/or instructions from your most recent vet visit in which your dog was diagnosed or treated for Chronic Kidney Disease (3 of 5). Upload a single document of any format below. You can upload additional pages/documents in the next question. Skip if finished.
16. Please upload medical records, receipts, and/or instructions from your most recent vet visit in which your dog was diagnosed or treated for Chronic Kidney Disease (4 of 5). Upload a single document of any format below. You can upload additional pages/documents in the next question. Skip if finished.
17. Please upload medical records, receipts, and/or instructions from your most recent vet visit in which your dog was diagnosed or treated for Chronic Kidney Disease (5 of 5). Upload a single document of any format below. You can upload additional pages/documents in the next question. Skip if finished.
